# Correction: Geographical epidemiology of *Hyalomma anatolicum* and *Rhipicephalus microplus* in Pakistan: A systematic review

**DOI:** 10.1371/journal.pone.0327736

**Published:** 2025-07-03

**Authors:** Abrar Hussain, Sabir Hussain, Ao Yu, Csaba Varga, Giulio A. De Leo, Rebecca L. Smith

The captions of Figs 1 and 2 are switched. The captions have been provided here:

**Fig 1:**
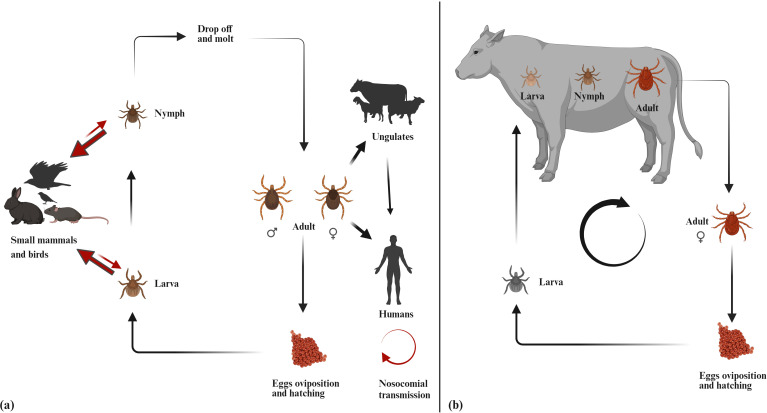
a) Life cycle of Hyalomma anatolicum (b) Life cycle of Rhipicephalus microplus (Figure made in BioRender.com).

**Fig 2:**
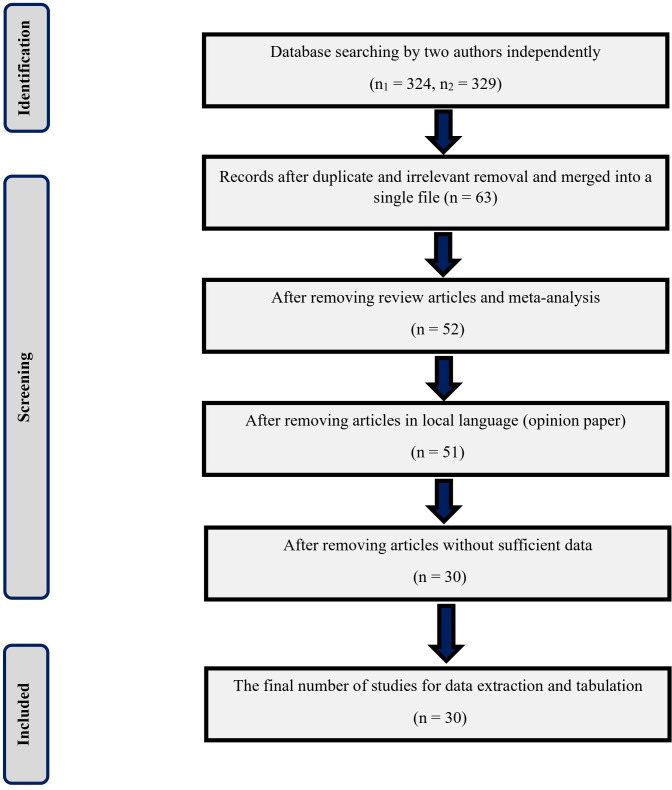
Schematic overview of the literature search procedure and results (PRISMA 2020 checklist is available as supporting information in S1 Checklist).
