# Supplementary material for: Geographical epidemiology of Hyalomma anatolicum and Rhipicephalus microplus in Pakistan: A systematic review
Source: PLoS One. 2024 Aug 23;19(8):e0309442. doi: 10.1371/journal.pone.0309442 (PMC11343457; doi:10.1371/journal.pone.0309442)
Supplement: Attachment — Submitted filename: Response to the Reviewer.docx [file pone.0309442.s006.docx]

**Response to the Reviewer**

**Editor**

1. **The geographical nature of each tick species distribution (like; desert, forest etc.....)**

Thank you!

A brief geographical distribution of both tick species has been added (Line #72-75)

2. **Photos for male and females of both ticks**

Thank you!

In the life cycle (Figure 2), images of both male and female ticks have been added.

3. **Stages of ticks on the ground due to *H. anatolicum* being multiple host while *R. microplus* is one host**

Thank you for your suggestion.

During data extraction, we recorded only the general tick numbers, disregarding their developmental stages, to ensure consistency across different districts. This approach was necessary because only a few papers provided detailed information on tick stages. (Line #128-130)

**Reviewer**

**Reviewer #1: Kindly check the grammatically error and the references carefully. The current manuscript is suitable for publication. This is meta analysis of prevalence CCHF and both species of ticks in Pakistan regions.**

Thank you for endorsing the manuscript for publishing. The manuscript has been revised by a native English speaker, and references have been formatted according to journal requirements.
